# Supplementary material for: Iterative improvement in the automatic modular design of robot swarms
Source: PeerJ Comput Sci. 2020 Dec 7;6:e322. doi: 10.7717/peerj-cs.322 (PMC7924708; doi:10.7717/peerj-cs.322)
Supplement: Supplemental Information 3 [file peerj-cs-06-322-s003.zip › argos3/doc/api/standalone/a00434_source.html]

ARGoS: plugins/robots/foot-bot/control\_interface/ci\_footbot\_base\_ground\_sensor.cpp Source File


- Main Page
- Related Pages
- Namespaces
- Classes
- Files

- File List
- File Members

# plugins/robots/foot-bot/control\_interface/ci\_footbot\_base\_ground\_sensor.cpp

Go to the documentation of this file.

```
00001 
00033 #include "ci_footbot_base_ground_sensor.h"
00034 
00035 #ifdef ARGOS_WITH_LUA
00036 #include <argos3/core/wrappers/lua/lua_utility.h>
00037 #endif
00038 
00039 namespace argos {
00040 
00041    /****************************************/
00042    /****************************************/
00043 
00044    CCI_FootBotBaseGroundSensor::CCI_FootBotBaseGroundSensor() :
00045       m_tReadings(8) {
00046       // Set the values for the base ground sensor offset (taken from the CAD model, in cm)
00047       m_tReadings[0].Offset.Set( 8.0, 0.0);
00048       m_tReadings[1].Offset.Set( 4.2, 6.5);
00049       m_tReadings[2].Offset.Set( 0.0, 8.0);
00050       m_tReadings[3].Offset.Set(-4.2, 6.5);
00051       m_tReadings[4].Offset.Set(-8.0, 0.0);
00052       m_tReadings[5].Offset.Set(-4.2,-6.5);
00053       m_tReadings[6].Offset.Set( 0.0,-8.0);
00054       m_tReadings[7].Offset.Set( 4.2,-6.5);
00055    }
00056 
00057    /****************************************/
00058    /****************************************/
00059 
00060    const CCI_FootBotBaseGroundSensor::TReadings& CCI_FootBotBaseGroundSensor::GetReadings() const {
00061      return m_tReadings;
00062    }
00063       
00064    /****************************************/
00065    /****************************************/
00066 
00067    std::ostream& operator<<(std::ostream& c_os,
00068                             const CCI_FootBotBaseGroundSensor::SReading& s_reading) {
00069       c_os << "Value=<" << s_reading.Value
00070            << ">, Offset=<" << s_reading.Offset << ">";
00071       return c_os;
00072    }
00073 
00074    /****************************************/
00075    /****************************************/
00076 
00077    std::ostream& operator<<(std::ostream& c_os,
00078                             const CCI_FootBotBaseGroundSensor::TReadings& t_readings) {
00079       if(! t_readings.empty()) {
00080          c_os << "{ " << t_readings[0].Value << " }";
00081          for(UInt32 i = 1; i < t_readings.size(); ++i) {
00082             c_os << " { " << t_readings[0].Value << " }";
00083          }
00084          c_os << std::endl;
00085       }
00086       return c_os;
00087    }
00088 
00089    /****************************************/
00090    /****************************************/
00091 
00092 #ifdef ARGOS_WITH_LUA
00093    void CCI_FootBotBaseGroundSensor::CreateLuaState(lua_State* pt_lua_state) {
00094       CLuaUtility::OpenRobotStateTable(pt_lua_state, "base_ground");
00095       for(size_t i = 0; i < m_tReadings.size(); ++i) {
00096          CLuaUtility::StartTable(pt_lua_state, i+1                            );
00097          CLuaUtility::AddToTable(pt_lua_state, "offset", m_tReadings[i].Offset);
00098          CLuaUtility::AddToTable(pt_lua_state, "value",  m_tReadings[i].Value );
00099          CLuaUtility::EndTable  (pt_lua_state                                 );
00100       }
00101       CLuaUtility::CloseRobotStateTable(pt_lua_state);
00102    }
00103 #endif
00104 
00105    /****************************************/
00106    /****************************************/
00107 
00108 #ifdef ARGOS_WITH_LUA
00109    void CCI_FootBotBaseGroundSensor::ReadingsToLuaState(lua_State* pt_lua_state) {
00110       lua_getfield(pt_lua_state, -1, "base_ground");
00111       for(size_t i = 0; i < m_tReadings.size(); ++i) {
00112          lua_pushnumber(pt_lua_state, i+1                 );
00113          lua_gettable  (pt_lua_state, -2                  );
00114          lua_pushnumber(pt_lua_state, m_tReadings[i].Value);
00115          lua_setfield  (pt_lua_state, -2, "value"         );
00116          lua_pop       (pt_lua_state, 1                   );
00117       }
00118       lua_pop(pt_lua_state, 1);
00119    }
00120 #endif
00121 
00122 
00123    /****************************************/
00124    /****************************************/
00125 
00126 }
```

---

Generated on 10 Jul 2018 for ARGoS by 
 1.6.1
